# Editorial: The bio-psycho-social approach to understanding mental disorders

**DOI:** 10.3389/fpsyg.2023.1225433

**Published:** 2023-06-15

**Authors:** Lu Hua Chen, Wilbert Law, Dorita H. F. Chang, Delin Sun

**Affiliations:** ^1^Department of Rehabilitation Sciences, Faculty of Health and Social Sciences, The Hong Kong Polytechnic University, Kowloon, Hong Kong SAR, China; ^2^Mental Health Research Center (MHRC), The Hong Kong Polytechnic University, Kowloon, Hong Kong SAR, China; ^3^Research Institute for Smart Ageing (RISA), The Hong Kong Polytechnic University, Kowloon, Hong Kong SAR, China; ^4^Department of Psychology, The Education University of Hong Kong, Tai Po, Hong Kong SAR, China; ^5^Centre for Psychosocial Health, The Education University of Hong Kong, Tai Po, Hong Kong SAR, China; ^6^Department of Psychology, The University of Hong Kong, Pokfulam, Hong Kong SAR, China

**Keywords:** bio-psycho-social model, mental disorders, depression, psychological distress, digital health technology

Mental disorders present considerable burdens to both individuals and society. Arriving at clinically meaningful treatments, however, is challenged by both high comorbidity among the various disorders, and the highly heterogeneous symptoms even within a specific disorder category. This reflects a reality that mental disorders are driven by multiple facets, including biological, psychological, and socio-environmental elements. Thus, a multi-dimensional approach involving biological, psychological, and socio-environmental factors affords a more comprehensive perspective to understand the mechanisms underpinning mental disorders (Figure 1) (Porter, 2020). Articles in this collection give a sampling of the interaction among these elements, and insight into how society may tackle the issues of mental health care moving forward.

**Figure 1:**
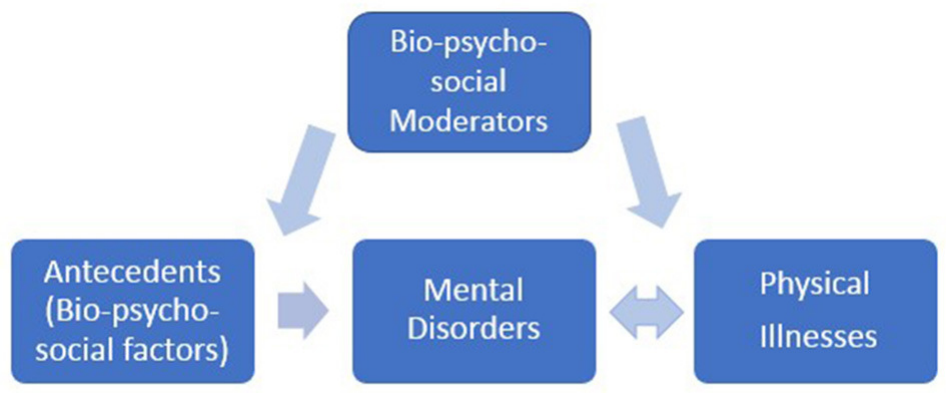
The bio-psycho-social model.

## Applying the bio-psycho-social model to understand depression

Depression is a mental disorder that can have a serious negative impact on how people feel, think, and behave. Extrinsic Emotion Regulation (EER) or providing support to others to regulate emotions, can reduce depressive symptoms such as stubborn thoughts and negative emotions. Massarwe and Cohen summarized previous work and argued that EER can enhance cognitive and affective processing that is impaired in depression and thus may be particularly beneficial for depressed patients. Their review of behavioral research shows that EER invokes processing related to emotion regulation, cognitive empathy, and reward, all of which are reduced in depression. Their review of neuroimaging findings suggests that these beneficial effects of the EER may be through the recruitment of brain regions associated with emotion regulation and stress reduction (e.g., the ventrolateral prefrontal cortex), regions involved in empathy (e.g., the medial prefrontal cortex) and reward-related regions (e.g., ventral striatum). The conceptual review by the two authors elucidates the mechanisms underlying the effectiveness of EER in patients with depression and thus offers new therapeutic avenues.

Marcu et al. examined the resting-state electroencephalogram (EEG)-derived frontal alpha asymmetry, which is one of the most popular electrophysiological markers used to identify depression. The authors investigated the association between resting-state EEG alpha asymmetries from multiple sites (frontal, extralateral, parietal) and multiple clinical indicators of depression in clinical populations. They report that alpha asymmetry is stronger in the parietal lobe than in the frontal and extralateral loci. They also found a moderately positive association between extra-lateral alpha asymmetry (eye closure only) and depression severity. However, they did not find significant differences in alpha asymmetry between different depression types. Therefore, the authors recommend further research on the relationship between depression and parietal and extralateral asymmetry indices in the future.

## Applying the bio-psycho-social model to understand how physical illness could be related to psychological distress

Psychological distress is often seen as having a strong association in the development of mental disorders (e.g., Connor et al., 2007; Yang et al., 2015). Historically, the study of distress focused on stressors and how each stressor exerts different degree of distress on people. An example would be Holmes and Rahe's Social Readjustment Rating Scale (SRRS; Holmes and Rahe, 1967). In the SRRS, different stressful life events would relate to a certain level of stress or Life Change Unit, e.g., major illness and sexual difficulties would have a value of 53 and 39, respectively. However, does all physical illness exert the same amount of stress to an individual? Would certain physical illnesses cause more stress and hence associate more with mental disorders? In this Research Topic, we collected two papers related to psychological distress and applied a bio-psycho-social factor model to understand the association between stress, mental health and physical illness.

In Bai et al.'s study, they compared the psychological stress and stress response from the Hypothalamus-pituitary-adrenocortical (HPA) and Autonomic Nervous System and symptoms of mental disorder between Chronic Prostatitis/Chronic Pelvic Pain Syndrome (CP/CPPS), CP/CPPS plus erectile dysfunction, erectile dysfunction alone, and healthy individuals. The rationale is that CP/CPPS is often associated with mental disorders. However, the reason of it could be due to an often associated condition, erectile dysfunction, which could be a bigger stressor. In this case, how each physical disorder would associate with what amount of stress could be delineated.

Additionally, some other associated questions regarding stress and mental disorder using the bio-psycho-social model include: Do the same stressor predicts mental disorders in different individuals in the same manner? What are some potential social factors (gender, sleeping duration, physical exercise) and physical factors (obesity and gender) that could buffer or exacerbate the impact of stressors? Are there bio-psycho-social factors that could affect individuals' experience of mental disorder which further influence their stress levels? Research findings from Linkas et al. have partially answered the above questions. In their study, they conducted a longitudinal study examining how inflammation, measured by C-reactive protein (CRP) and transforming growth factor-alpha (TGF-α), would affect development of psychological distress in adolescents. In addition, they examined bio-psycho-social factors such as gender, sleep duration, physical exercise and obesity as moderating factors that could buffer or exacerbate the impact of inflammation on adolescents. They found that all these factors play important roles in inflammation responses which result in different levels of psychological distress 2 years later.

In both collected papers, authors have used psychological (self-report) method together with biological method (hormones, heart-rate variability, pro-inflammatory markers in the blood) for their investigations. These methods yield divergent or convergent results and could afford important information to better understand the diversities of developing outcomes in mental disorders (Caparros-Gonzalez et al., 2022).

## Conclusions and future directions

Our collection of papers also highlights utilization of modern digital health technology in mental health and illustrate some concerns. The review by Varela-Moreno et al. which considered the comorbid relationship between diabetes mellitus and depression, suggests that eHealth treatments appear to benefit depressive symptomatology while not to benefit glycemic control. The authors note that much remains to be done here, as all studies to date using eHeatlh/eDelivery are methodologically heterogeneous, and the feasibility and economics of using such interventions via their effectiveness remain to be established.

In a similar vein, Uchmanowicz et al. considered digital health solutions for patients with heart failure. The thought is that offering a suite of interactive tele-feedback and/or real time services, via mobile apps or the WWW more broadly, can better address bio-psycho-social variables that may act in concert to support both physical and psychological wellbeing. Consistently, early studies appear to indicate some benefits to secondary outcomes of heart failure (depression, fatigue).

While not perfect, the benefits of digital health technology to improving patients' quality of life, both as outlined by Uchmanowicz et al. and Varela-Moreno et al. cannot be understated. It should be clear that the path forward in tackling mental health care must involve creative integration of modern technologies. The exact suite of tools that produce the best compromise in efficacy, accessibility, and economics awaits deserves empirical attention.

In summary, our collection of papers contribute to a better understanding of the potential mechanisms underpinning mental disorders and highlight the promising role of digital health technology in supporting mental health patients in the future.

## Author contributions

All authors listed have made a substantial, direct, and intellectual contribution to the work and approved it for publication.
